# Epidemiological Evidence on the Associations of Metal Exposure with Alzheimer’s Disease and Related Dementias Among Elderly Women

**DOI:** 10.3390/jcm14113776

**Published:** 2025-05-28

**Authors:** Fahmida Rashid, Khalid M. Khan, Samyukthaa Saiprakash, Giasuddin Ahmed, Rasheda Sultana, Faruque Parvez, Zhahirul Islam, Md. Shiblur Rahaman

**Affiliations:** 1 Department of Public Health, College of Health Sciences, Sam Houston State University, Texas, TX 77341, USA; fxr040@shsu.edu (F.R.); mxs218@shsu.edu (R.S.); 2 College of Osteopathic Medicine, Sam Houston State University, Texas, TX 77304, USA; sxs232@shsu.edu; 3 Department of Biology and Chemistry, Texas A & M International University, Laredo, TX 78041, USA; giasuddin.ahmed@tamiu.edu; 4 Department of Environmental Health Sciences, Mailman School of Public Health, Columbia University, New York, NY 10032, USA; mp844@cumc.columbia.edu; 5 Gut-Brain Axis Laboratory, Infectious Diseases Division (IDD), Bangladesh (icddr,b), Dhaka 1212, Bangladesh; zislam@icddrb.org; 6 Department of Environmental Science and Disaster Management, Noakhali Science and Technology University, Noakhali 3814, Bangladesh

**Keywords:** Alzheimer’s disease, dementia, ADRD, heavy metals, elderly, women, epidemiological study

## Abstract

**Background:** Emerging evidence suggests a potential link between heavy metals and Alzheimer’s disease and related dementias (AD/ADRD). This study compiled epidemiological evidence from research published over the past 11 years on the impact of metals on AD/ADRD in women. Women have unique risk factors for late onset of AD/ADRD, in addition to genetic factors, apolipoprotein E allele (APOE4), and longer life expectancy. Furthermore, women are twice likely as men to experience depression, which increases their risk of developing AD/ADRD. Our narrative review underscored the necessity of a sex-specific approach to address women’s vulnerability to AD/ADRD. **Methods:** Electronic databases, including PubMed, Google Scholar, NIOSH Toxline, and Scopus, were thoroughly searched to identify primary epidemiological studies on older women exposed to metals and published between 2014 to 2024. **Results:** We identified 34 epidemiological studies that met the inclusion criteria. The findings revealed a complex interplay between environmental metals such as lead (Pb), cadmium (Cd), arsenic (As), manganese (Mn), selenium (Se), iron (Fe), zinc (Zn), copper (Cu), magnesium (Mg), and calcium (Ca) and the risk of AD/ADRD in women. Significant adverse effects were reported for Cu, Cd, As, Pb, and Mn while significant protective effects were found between Se, Fe, and Zn in blood and AD/ADRD among older women. However, some studies also reported no correlations. **Conclusions:** Overall, our review identified contrasting results regarding the effects of metals on AD/ADRD in women. Future studies should collect additional evidence to understanding the effects of heavy metals on AD/ADRD in women for developing preventive measures.

## 1. Introduction

Alzheimer’s disease and related dementias (AD/ADRD) are the most prevalent neurocognitive diseases and recognized as a pressing public health challenge worldwide. Currently, approximately 57 million people worldwide suffer from AD/ADRD [1,2] which will continue to increase in the coming years. In the United States (U.S.), more than 5.5 million people have AD/ADRD, a number projected to reach 8.5 million by the end of 2030 [3]. Alzheimer’s-related dementia (ADRD) burden varies by region. East Asia has the highest number of individuals with dementia (9.8 million), followed by Western Europe (7.5 million), South Asia (5.1 million), and North America (4.8 million) [4]. Surprisingly, high-income countries (HICs) tend to show higher rates of AD/ADRD prevalence. Recent trends indicate a significant increase in the incidence of dementia (mostly ADRD) in low- and middle-income countries (LMICs). Currently, 60–67% of people with dementia live in LMICs, and 71% of new diagnoses are expected to be from these LMICs [5,6]. LMICs in Asian countries, including India and China, are expected to show over 300% increase in people with dementia between 2001 and 2040, much higher than the high-income countries (100% increase) within the same timeframe [7,8].

Social, biological, and environmental risk factors are responsible for the growing concerns over AD/ADRD. Genomic factors that contribute to the likelihood of AD/ADRD include APOE4 [9]. At the same time, emerging evidence suggests a potential link between various social and environmental factors and ADRD [10]. For instance, higher educational levels support cognitive performance and lower the risk of dementia [11]. Furthermore, living in a green area, such as parks and gardens, has positive impacts on cognitive function [12]. Conversely, long-term exposure to air pollution has been associated with an increased risk of dementia [13]. Emerging research highlights the role of exposome factors, such as workplace environmental exposure [14] and heavy metals influencing dementia risk and contributing to health disparities [10]. Among environmental factors, metal exposure remains an understudied aspect of the exposome, particularly in older women. Metal exposure may play a significant role in increasing the risk of cognitive impairment, leading to AD/ADRD [1,10,15,16,17,18,19].

Among aging populations at risk of developing AD/ADRD, older women (over 50 years old) represent a particularly vulnerable group [20,21]. Several risk factors put women at higher risk. Women live longer than men, making them more likely to reach an age to develop AD/ADRD [22]. Additionally, women account for almost two-thirds of AD dementia cases [23]. Research has shown that over 65% of those diagnosed with late-onset dementia are women [24]. Furthermore, women are more likely to experience certain risk factors, such as a higher prevalence of depression [25], which is a known risk factor for ADRD [26]. The vulnerability of women is further exacerbated by historical and sociological circumstances, including their historically limited access to education in many developing countries [27,28]. Other factors, such as genetic predispositions and female-specific conditions including early menopause and sleep apnea, also make women highly susceptible to AD/ADRD [27,29]. Despite this increased susceptibility, much remains unknown about the precise effects of environmental chemicals, particularly heavy metal exposure, on AD/ADRD in older women. Metal exposure through food, soil, air, and drinking water is common in both developed and developing countries due to increased industrial activities and natural contamination (i.e., geogenic sources). In regions where industrial activities involve toxic metals, there are increased risks to nearby populations. Pollution not only directly affects populations but contaminates waterways and aquatic life, which is an essential food source utilized by these populations. Additionally, pollution affects vegetation that relies on these contaminated sources [30].

Highly toxic metals, especially lead (Pb), cadmium (Cd), and arsenic (As), along with essential elements such as manganese (Mn), selenium (Se), iron (Fe), zinc (Zn), copper (Cu), magnesium (Mg), and calcium (Ca) are associated with biological alterations that may increase the risk of AD/ADRD related symptoms [31,32,33,34,35,36,37]. For instance, Pb affects several symptoms indicative of cognitive decline, including memory loss, decreased brain signaling, and a reduced capacity for learning [38]. Cd can cross the blood–brain barrier (BBB) and contribute to inflammation, oxidative stress, and ultimately neuronal apoptosis, all of which are dysregulated processes in neurodegenerative conditions [15]. Exposure to Cd has been linked to dementia-related learning and memory impairments as well as mood disorders. Essential elements such as Fe, Cu, Zn, and Ca are necessary for brain development, neurotransmitter synthesis, immune system function, and cellular homeostasis [34]. Deficiencies or excesses of Mn, Se, and Mg may reduce brain functions, as these minerals play key roles in antioxidant processes and proper neuronal transmission [35,39,40]. Overall, an imbalance of any of these micronutrients and metals may lead to neurodegenerative diseases, including AD/ADRD. Maintaining the homeostasis of these metals is more crucial, particularly in older women. For example, Mn neurotoxicity is influenced by other trace metals, especially Fe, as they share common transport pathways. A deficiency in Fe can increase the Mn level [41], potentially leading to toxicity at even moderate doses. Since Fe deficiency is very common among women [42], this homeostasis interplay is particularly relevant in older women, as hormonal fluctuations and metabolic differences influence metal absorption and regulation [43].

Despite increasing research on cognitive impairment, dementia, and other AD/ADRD outcomes or symptoms, significant gaps remain in exploring how sex-specific factors such as genetic composition, hormonal alterations, metabolism of metals, and environmental factors can uniquely influence women. While some studies have explored the sex influence on cognitive function, very few specifically focus on women, particularly in relation to metal exposure. The existing research includes a very limited number of women AD participants compared to men. Additionally, many studies do not conduct comparative analysis, making it difficult to draw conclusion about sex-specific effects. Furthermore, most research is cross-sectional, making it difficult to establish causality between the metal exposure and cognitive impairment, especially in women; therefore, it is crucial to critically evaluate the current state of knowledge regarding the roles of both toxic and nutritional metals in either elevating or reducing the risk of AD/ADRD in women.

We thus conducted a comprehensive narrative review of the available epidemiological studies with the goal of improving our understanding of the effects of heavy metals on AD/ADRD among older women.

## 2. Methods

An extensive search of electronic databases (PubMed, Google Scholar, NIOSH Toxline, and Scopus) was conducted to identify peer-reviewed articles from indexed journals on epidemiological studies investigating the relationship between heavy metals (e.g., Zn, Fe, Mg, Ca, Se, Pb, As, Mn, Cd, and Cu) and AD/ADRD. The time frame for selecting epidemiological articles was also restricted to within the past 11 years (i.e., January 2014–September 2024). Studies that were not written in English, not indexed, not available online, and non-peer-reviewed, such as manuscripts, reports, articles from conferences, or doctoral dissertations, were excluded from this review. Another major selection criterion was to include peer-reviewed epidemiological articles that reported AD/ADRD outcomes in women only or both male and female participants over 50 years of age. Studies were excluded if they did not involve AD/ADRD, dementia, and cognitive impairment; were not original research (e.g., reviews) or were abstracts; were animal trials; were male trials; or did not present sex-based results.

We used a combination of the following keywords: “humans” or “patients” or “sub-jects” or “participants” or “person” or “population” or “individuals” and “elderly” or “aged” and “women” or “female” and “heavy metals” or “trace elements” or “toxic metals” or “nutritional metals” or “lead (Pb)” or “cadmium (Cd)” or “arsenic (As)” or “manganese (Mn)” or “selenium (Se)” or “iron (Fe)” or “zinc (Zn)” or “copper (Cu)” or “magnesium (Mg)” or “calcium (Ca)” and “dementia” or “Alzheimer” or “Alzheimer’s related dementias” or “cognitive impairment” or “mild cognitive impairment” or “cognitive dysfunction”.

**Figure 1:**
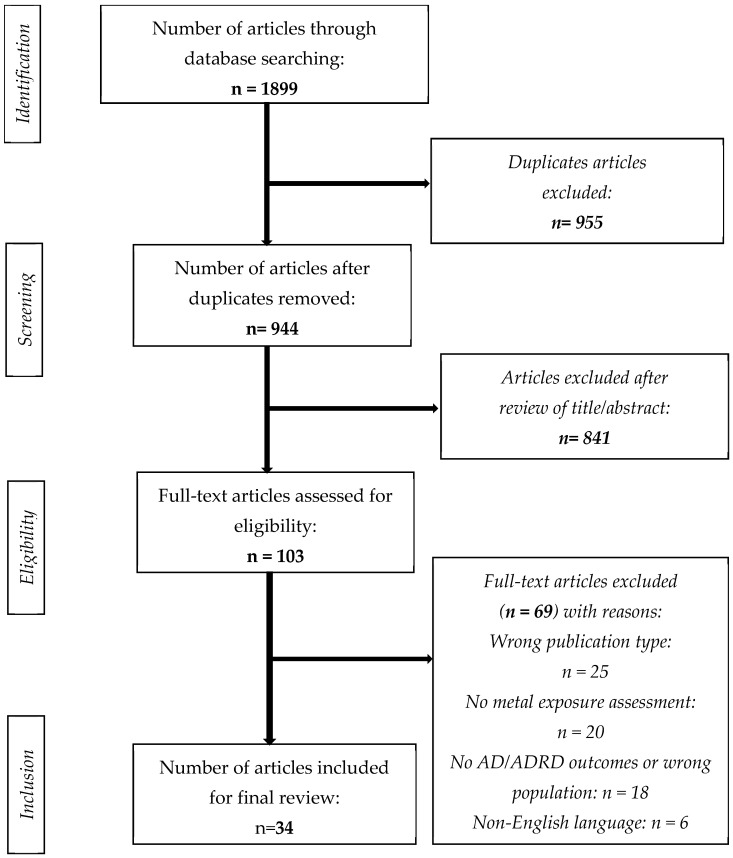
Flow chart of the search strategy.

In total, 1899 articles were identified in the preliminary search. Next, 955 duplicates were removed, and 944 articles were screened by title and abstract. Then, 841 articles were excluded because they did not fulfill the inclusion criteria. Full-text screening of 103 articles resulted in the exclusion of 69 articles based on the following reasons: inappropriate publication type (n = 25), lack of metal exposure assessment (n = 20), absence of AD/ADRD outcomes or inappropriate population (n = 18), and non-English language (n = 6). Finally, 34 peer-reviewed and full-text published articles were included for analysis in this review. Figure 1 illustrates the search strategy for epidemiological studies and stepwise selection of articles for the current review.

## 3. Results

### 3.1. Adverse Effects of Metals on AD/ADRD Outcomes Among Women

#### 3.1.1. Lead (Pb)

In this review, five articles were identified that described the adverse effects of Pb, and among them, some studies found that the relationship between Pb and cognitive function also had significant sex differences (Table 1). A study by Power et al. (2014) [17], conducted on women, identified long-term cumulative exposure to Pb to be associated with cognitive decline. In this cohort study, they measured bone Pb (micrograms Pb per gram bone mineral) among 584 women using a validated telephone-based cognitive assessment [17].

Another cohort study among 1148 individuals (55.23% women) by Liu et al. (2024) [44] identified a stronger positive association between blood Pb and cognitive impairment (CI) (OR= 1.79; 95% CI: 1.11–2.88, *p* = 0.016), with sex being significant. Older adults with CI were measured via the Chinese version of the Mini-Mental State Examination (MMSE) score for cognitive assessment (lower score indicated poor cognitive function). Most CI individuals tended to be women (25.39%) compared to men (15.95%) [44]. Similarly, Qing et al. (2024) [16] found that blood and urinary Pb levels were significantly and inversely correlated with the Montreal Cognitive Assessment (MoCA) score. Overall, the sex difference was significant (*p* < 0.05) in Ying’s study. In another study, Fu et al. (2024) [45] addressed the dose–response relationship between blood Pb and a cognitive function test called the Digit Symbol Substitution Test (DSST). The female group with a higher blood Pb level (2.09 µg/dL) was more likely to have worse performance than their male counterparts.

On the other hand, Fathabadi et al. (2018) [38] found a significantly higher blood lead level (BLL) among the AD patients (BLL, OR = 1.05, 95% CI: 1.01–1.09, *p* = 0.01).

**Table 1:** Summary of Epidemiological Studies.

Author and Metal(s)	Geographical Origin	Age (Mean Years ± SD)	Sample Size (Female)
**Study design: Cohort**			
Ashraf et al. (2019) [46], Fe and Zn	United Kingdom	77.5± 6.2	88 (48)
Giacconi et al. (2019) [47], Cu and Zn	Italy	77.5 ± 0.05	179 (132)
Gong et al. (2021) [34], Fe	United States	78 ± 8.6	3131 (1642)
Rembach et al. (2014) [48], Zn	Australia	78.8 ± 8.6	1084(634)
Jinhui Yu et al. (2023) [49], Se and Zn	China	71.70 ± 6.38	1025 (548)
Lui H et al. (2021) [15], Cd	China	73.79 ± 5.92	1554 (994)
Liu Q et al. (2024) [44], Pb	China	71.14 ± 5.78	1148 (634)
Power et al. (2014) [17], Pb	United States	61 ± 6	584 (584)
Min J et al. (2016) [50], Cd	United States	74.5 ± 8.36	4064 (2032)
Peng Q et al. (2017) [51], Cd	United States	71.1 ± 0.24	2023 (971)
**Study design: Case–control**			
Koseoglu et al. (2021) [52], As and Se	Turkey	75.8 ± 5.8	80 (47)
Cardoso et al. (2017) [53], Se	Australia	82.1 ± 1.2	209 (40)
Fathabadi et al. (2018) [38], Pb	Iran	68.65 ± 7.39	81 (36)
Gu et al. (2021) [54] As and Se	China	71.8 ± 6.5	1066 (583)
Huang et al. (2022) [55], Cd	United States	≥60 years old	1918 (964)
Jouini et al. (2021) [56], Fe and Ca	Tunisia	70.538 ± 7.57	167 (102)
Zhang et al. (2022) [18], Cu and Cd	China	67 ± 4.0	1667 (956)
Koc et al. (2015) [57], Cu, Mn, Mg, Se, and Mg	Turkey	77.66 ± 9.28	78 (39)
Li H et al. (2018) [58], Cd	United States	69.14 ± 10.91	2068 (1126)
Park et al. (2019) [59], Fe	Korea	69.2 ± 8.4	127 (100)
Rozzini et al. (2018) [60], Cu	Italy	77.1 ± 7.6	108 (58)
Socha et al. (2021) [36], Cu, Se, and Zn	Poland	67.0 ± 7.9	170 (126)
Sternberg et al. (2017) [61], Fe	United states	70.6 ± 7.4	85 (36)
Xu et al. (2018) [62], Mg, Ca, Fe, Cu, Zn, and Se	England	78.2 ± 1.3	84 (39)
Yang et al. (2018) [33], As and Se	China	76.48 ± 7.41	434 (244)
**Study design: Cross-sectional**			
Cheng et al. (2022) [63], Se	China	78.5 ± 10.68	3813 (1925)
Duhan et al. (2024) [64], Se and Cu	China	72.4 ± 5.3	416 (208)
Hare et al. (2016) [65], Mn	Australia	78 ± 8.6	100 (436)
Nascimento et al. (2021) [35], Se	Brazil	74.41 ± 7.1	102 (70)
Wang et al. (2024) [66], Se and Cd	United States	69.16 ± 6.68	1460 (675)
Wang X et al. (2021) [19], As	China	57.0 ± 11.5	1556 (754)
Wang et al. (2024) [67], As, Fe, and Zn	China	97.10 ± 4.92	408 (322)
Qing et al. (2024) [16], Pb and Cd	China	70.55 ± 6.3	836 (485)
Zixuan Fu et al. (2024) [45], Pb, Cu, and Cd	United States	≥60	811 (421)

#### 3.1.2. Cadmium (Cd)

In this review, we found nine studies that showed a positive association (indicating negative effects of a specific metal) between Cd exposure and AD/ADRD outcomes (Table 1). A case–control study performed by Huang et al. (2022) [55] with a total of 1918 participants (964 female and 467 low cognitive performance) found high-level blood Cd associated with a higher risk of CI after adjusting for age and gender (OR = 1.558, 95% CI:1.144–2.123; *p* = 0.006). However, they did not find any sex-specific risk differences [55]. Similarly, a significant positive correlation between plasma Cd (OR = 1.456, 95% CI: 1.003–2.114, *p* = 0.048) and the risk of CI was reported by Zhang et al. (2022) in a cross-sectional study where women showed higher CI [18]. In China, a prospective cohort study conducted by Liu H et al. (2021) found higher blood Cd significantly associated with greater cognitive decline, with women showing higher risk that was statistically significant (994 were female participants among 1554 total study participants) [15]. A case–control study by Li H et al. (2018) involving 2068 American participants (1126 female) found that a higher level of blood Cd was associated with worse cognitive function (β = −0.14, 95% CI −0.25 to –0.03; *p* < 0.0001) [58].

Min et al. (2016) conducted a cohort study with 4064 participants (50% female participants), where female participants had higher blood Cd levels, and high blood Cd was significantly associated with the risk of AD mortality [50]. Similar findings were reported in another cohort study conducted by Peng et al. (2017) [51].

#### 3.1.3. Arsenic (As)

In this review, four articles were identified showing positive relationships (adverse effects) between As exposure and AD/ADRD-related outcomes (Table 1). A case–control study conducted in Turkey among 80 individuals (more than 50% female participants) showed a significant positive correlation between As levels (in both hair and nail samples) and AD outcomes (*p* < 0.001), although the researchers did not find any association between As levels and sex [52]. Another case–control study involving 1066 Chinese older adults revealed that As exposure was associated with an increased risk of cognitive dysfunction (As, OR = 2.06 (95% CI: 1.30–3.25; *p* = 0.002) as evaluated by MMSE, and sex was not associated with blood As concentration [54].

Wang and colleagues investigated the association between hair As exposure and cognitive impairment among 1556 individuals (more than half women participants) and assessed participants’ cognitive function using MMSE. They showed that female participants had a significantly higher prevalence of CI [19]. A case–control study conducted by Yang et al. (2018) demonstrated that female participants were more likely to develop and significantly at risk for developing AD compared to the male participants [33]. The Clinical Dementia Rating (CDR) scale, along with the MMSE, was used to assess cognitive function. This study also reported that urinary As is associated with the increased risk of AD [33].

#### 3.1.4. Multiple Metals (Adverse Effects and/or No Effects)

In this review, it was found that several papers examined the effects of more than one metal on AD/ADRD in the same sample of participants (Table 2).

Cu has been consistently associated with AD progression. Rozzini et al. (2018) [60], in a case–control study conducted among 108 Italian participants (58 females), reported female AD patients with MCI and dementia had higher total serum Cu levels compared to male participants (1147.7 µg/L ± 200.4 vs. 1060.2 µg/L ± 219.0; *p* = 0.016) [60]. In another study, Cu showed a positive correlation with the risk of cognitive performance (OR = 1.519, 95% CI: 1.050–2.197, *p* = 0.026). Additionally, the author mentioned participants with CI tended to be women (*p*< 0.001) [18].

Duan et al. (2024) found that higher levels of Ca were associated with lower cognitive function (β = −0.67, 95% CI −1.26 to –0.8; *p* = 0.25) [64]. Socha et al. (2021) [36] investigated if Cu or the Cu/Zn molar ratio had any effects on AD patients. In this case–control study, they found a non-significant higher level of Cu among women AD participants (1.07 ± 0.23 vs. 0.90 ± 0.18). Additionally, the Cu/Zn molar ratio was significantly higher among females with AD (1.59 ± 0.45 in females versus 1.31 ± 0.48 in males; *p* < 0.001) [36]. Although Mn is an essential element, elevated levels have been linked to cognitive dysfunction. Koc et al. (2015) observed significantly higher hair Mn levels in AD patients compared to healthy controls, though no direct sex differences were noted [57]. However, the unique susceptibility of women to neurotoxic effects due to differences in Mn metabolism and hormonal regulation remains an area requiring further exploration [68]. Dysregulated Ca homeostasis has been implicated in neurodegenerative processes associated with AD. Wang et al. (2024) [67] demonstrated a positive correlation between plasma Ca levels and cognitive impairment risk, though no significant association was observed in women specifically. Despite this, previous research indicated that Ca dysregulation, potentially influenced by estrogen deficiency postmenopause, could uniquely affect women’s neural Ca signaling pathways, amplifying their vulnerability to AD-related cognitive decline [67,69].

Zn, primarily considered a nutritional metal, is found in higher amounts among AD patients. Yu et al. (2023) reported that a higher whole-blood Zn level increased the risk of MCI (OR: 1.87, 95% CI: 1.131–3.101; *p* = 0.015), especially among females [49]. Similarly, Ashraf et al. (2019) [46] reported higher Zn levels in AD patients. However, they did not report any sex-based comparison [46]. Two studies found that a high level of Se was associated with increased risk of CI (OR = 1.947, 95% CI 1.20–3.17; *p* = 0.007) [54] and AD (OR = 2.33; 95% CI: 1.13–4.81; *p* < 0.05) [33]. 

Park et al. (2019) conducted a retrospective observational study in Korea among 127 participants to observe the effects of Fe on AD progression. The authors reported higher plasma Fe levels in the motor cortex among AD patients [59]. Two epidemiological studies examined Mg to observe its effects on AD/ADRD, but they did not find any correlation [57,62].

### 3.2. Beneficial Effects of Metals on AD/ADRD Outcomes Among Women

#### 3.2.1. Zinc (Zn)

Several studies have discussed the relationship between Zn and AD/ADRD (Table 3). Giacconi et al. (2019) [47] and Koc et al. (2015) [57] found that overall Zn levels were significantly lower among AD patients compared to the healthy group, although no sex-specific differences were noted. Similarly, in a cross-sectional study among 408 participants, Wang et al. (2024) [67] reported a negative correlation between Zn and the risk of CI.

Rembach et al. (2014) [48], in a longitudinal prospective Australian cohort study, reported serum Zn to be non-significantly lower in women (11.910 ± 2413; vs. 12.706 ± 3.332; *p* = 0.073). The same research also found lower MMSE scores among AD patients compared to the healthy group (*p* < 0.001). Socha et al. (2021) [36] conducted a case–control study in Poland with 170 participants and reported significantly lower serum Zn levels in AD patients. Notably, female AD patients had even lower Zn levels than men (0.71 ± 0.14 vs. 0.84 ± 0.61 male; *p* < 0.05). Xu Jingshu et al. (2018) conducted a case–control study in England, which reported non-significantly lower levels of serum Zn among the female participants [62].

#### 3.2.2. Selenium (Se)

The relationship between AD/ADRD and cognitive function with Se level has been widely assessed because of its significant effects on brain homeostasis maintenance [69]. Several epidemiological studies have examined Se level with respect to AD/ADRD and cognitive function, providing insight into its sex-specific outcomes (Table 3). Cardoso et al. (2017) [53] and Koc et al. (2015) [57] conducted case–control studies. Both studies reported no significant sex differences as far as Se levels were concerned, although the overall reduction in Se levels in AD patients indicated a possible protective role (*p* < 0.005). Wang et al. (2024) [66] and Duan et al. (2024) [64] performed two cross-sectional studies among 1460 and 406 participants, respectively. Both studies found protective effects of Se on cognitive function (β = 0.049, 95% CI: 0.022, 0.076; *p* < 0.05), (β = 0.32, 95% CI: 0.09, 0.55; *p* = 0.007); however, neither study included any sex-based comparison to detect the Se level among male and female AD patients.

Cheng et al. (2022) [63] examined the association between urinary Se levels and cognitive performance in a large cross-sectional study of 3814 individuals in China that included 1925 female participants. Se levels were positively associated with MMSE scores in both single-element (β = 0.26; 95% CI: 0.01–0.52) and multi-element models (β = 0.31; 95% CI: 0.02–0.60) after covariate adjustment. Women showed significantly lower MMSE scores (*p* < 0.001) compared to males [63], and Yu et al. (2023) [49] provided further evidence of sex-specific vulnerabilities. In their cohort study of 1025 participants in China, lower Se levels were observed in individuals with MCI, with a significant association identified in women (OR = 0.33; 95% CI: 0.193-0.591; *p* < 0.05) [49].

In a case–control study from Poland, Socha et al. (2021) [36] provided one of the few studies with explicit sex comparisons. They reported significantly lower serum Se levels in AD patients, with female AD patients exhibiting even lower levels than males (female: 68.8 ± 19.8; male: 69.5 ± 17.9; *p* < 0.05) [36].

#### 3.2.3. Multiple Metals (Beneficial Effects)

Our review found several epidemiological investigations examining the effects of more than one metal on AD/ADRD in the same population (Table 3). For instance, Xu et al. (2018) [62] conducted a case–control study in England reporting a non-significant lower serum Fe among women with AD/ADRD compared to men [62]. Ashraf et al. (2019) [46] identified lower plasma Fe levels in AD patients in a cohort study among 88 participants in the United Kingdom [46]. However, they did not perform any sex-based comparison. The findings of the study emphasize the importance of monitoring Fe levels in women, particularly during menopause, which may reduce the risk of AD and related cognitive impairment. Gong et al. (2021) [34] conducted a similar cohort study in Italy in a larger sample (3131 participants). They reported an inverse dose–response relationship between Fe and cognitive dysfunction [34]. Hare et al. (2016) [65] observed reduced serum Mn levels in AD patients (*p* < 0.05), with lower erythrocyte Mn levels trending among women [65].

## 4. Discussion

Evidence from various epidemiological studies demonstrated potential positive and negative effects of metals on AD/ADRD in women. Neutral effects of metals were also observed in several epidemiological investigations. Toxic metals such as As, Pb, Cu, and Cd were associated with increased risk of AD/ADRD in women. Higher Zn levels repeatedly and consistently showed protective effects against the development of AD/ADRD. Evidence of lower levels of Zn in female AD patients compared to their male counterparts indicated a possible role of Zn deficiency in elevating the risk of AD/ADRD-related outcomes in women. Higher Se levels also showed mostly positive impacts. Fe and Mn showed beneficial association with AD/ADRD outcomes, with women showing greater vulnerability to deficiencies. Surprisingly, some nutritional metals, such as Zn and Fe, also demonstrated detrimental effects on AD/ADRD because the effects of metals depend on the level of exposure [70].

### 4.1. Adverse Effects of Metal Exposure on AD/ADRD

Neurotoxic metal Pb is associated with various cognitive declines, including learning, memory, working memory, attention, and executive function [71,72,73,74]. In this review, we found significantly higher BLL among the AD patients [38]. However, higher BLL was demonstrated among men, indicating a possibly lower risk in women. Some factors may have influenced the findings in opposite directions, including the small sample size (n = 81), the case–control study design, and the demographic factors (diet, income, and other social stressors). Prolonged life exposure to Pb is well documented as a neurotoxin and linked to cognitive decline in later life [75], and mild cognitive impairment (MCI) is considered an early stage of AD/ADRD [45].

Cd is a well-known toxic metal, and numerous researchers have studied its relationship with the etiology of AD/ADRD across the globe. Excessive amounts of Cd have been found in the liver, plasma, and brain tissue among AD patients [76,77,78]. In this review, several studies were identified that reported the adverse effects of Cd on AD/ADRD. Additionally, the effects on women were also reported. After adjusting for smoking status, women had significantly higher blood Cd compared to men [50]. Recent studies have also established a link between high Cd concentration and a decline in cognitive functions [16,45,66]. Although the majority of the studies found associations between Cd and CI, a case–control study among Taiwanese AD patients conducted by Yang et al. (2018) did not identify any association between Cd exposure and cognitive decline using propensity score matching [33]. Women are particularly vulnerable to the neurotoxicity of Cd based on hormonal and physiological factors. Reduced iron storage in women, especially post-menopausal women, enhances gastrointestinal Cd absorption, leading to enhanced body burden [79].

As is well studied for its neurotoxicity properties. Although sex differences were not uniformly observed across studies, some reported higher risk in female subjects [19,33]. Mechanistic pathways such as oxidative stress, mitochondrial dysfunction, and inflammation caused by chronic As exposure may increase the risk among female populations. Previous studies showed that low levels of arsenic in water could be directly correlated with myocardial infarction risk. Furthermore, supportive vascular insufficiency and myocardial infarction can result in brain hypoperfusion, white matter lesions, and infarctions of the brain, leading to cognitive decline and eventually AD/ADRD [80].

Interestingly, a non-significant higher level of Cu and a significantly higher level of Cu/Zn molar ratio among women AD participants were reported by Socha et al. (2021) [36]. These findings are clinically important, as an elevated Cu/Zn ratio is recognized as a biomarker of oxidative stress [81]. Furthermore, disproportionately low Zn and elevated Cu may compromise the antioxidant functionality of various enzymes [82]. Women are more vulnerable to developing neurocognitive issues due to differences in antioxidant capacity and hormonal regulation [83,84,85].

A previous study reported that higher levels of Ca are associated with lower cognitive function [64]. The main mechanism may be attributed to the disparity between intracellular and extracellular Ca^2+^ homeostasis. High blood Ca^2+^ crosses the blood–brain barrier [86] and raises extracellular Ca^2+^, stimulating Ca-sensing receptors, augmenting Ca^2+^ entry [87], closing voltage-sensitive Ca channels, and raising membrane resistance [88]. This disrupts Ca^2+^ homeostasis, triggering neuronal death [89,90] and dementia lesions. Despite this, previous research indicated that Ca dysregulation, potentially influenced by estrogen deficiency post menopause, could uniquely affect women’s neural Ca signaling pathways, amplifying their vulnerability to AD-related cognitive decline [91].

Although Mn is an essential element, elevated levels have been linked to cognitive dysfunction. However, the unique susceptibility of women to neurotoxic effects due to differences in Mn metabolism and hormonal regulation remains an area requiring further exploration [68].

While Zn is an essential micronutrient, excessive Zn, particularly at whole-blood concentrations, can potentially be detrimental to women with MCI and AD [46,49]. This may be due to sex-specific differences in hormonal homeostasis, metal transport, and neuroinflammation [92,93]. Higher plasma Fe levels in the motor cortex among AD patients was reported by a study conducted by Park et al. (2019) [59]. However, several studies highlighted Fe accumulation as an emerging biomarker and a potential modulator of disease progression in various neurodegenerative diseases, including AD, especially among women [94,95,96,97].

### 4.2. Neuroprotective and Essential Roles of Metals

As the data indicate, women generally have lower levels of Zn. Consequently, the prevalence of AD was found to be higher among Zn-deficient women [98], and they tend to show more CI than men [99]. One possible reason for the low-Zn status is that women tend to eat less meat, which is rich in Zn and quickly absorbed by the body, and prefer foods like beans, which contain substances that can reduce the body’s ability to absorb Zn [100]. Additionally, women are more sensitive to Zn deficiency due to the effects of the female hormone estrogen [101].

Our review highlights the significantly lower serum Se levels in AD patients, with female AD patients exhibiting even lower levels than males [36]. Se levels were shown to be positively associated with MMSE scores and found a dose–response relationship between urinary Se and MMSE score and cognitive performance. Significantly lower MMSE scores were found in women, and these findings underscore more cognitive decline in women [63], possibly due to the differences in Se metabolism between men and women [69]. Results suggest that women may be disproportionately affected by Se deficiency, potentially contributing to their heightened risk of developing AD/ADRD. Previous studies have shown Se levels to be positively correlated with cognitive function, and a deficiency in Se can result in cognitive impairment [102]. Overall, the findings indicate that women may be more vulnerable to Se deficiency, which eventually increases the risk of AD/ADRD-related outcomes among them [103,104].

Our review identified lower plasma Fe levels in AD patients [46]. Gong et al. (2021) reported an inverse dose–response relationship between Fe and cognitive dysfunction, i.e., indicating CI increased as serum Fe level decreased [34]. A negative correlation between Fe and CI was also reported in a cross-sectional study in China [67]. Neither of these studies showed any sex-specific findings. The findings of the study emphasize the importance of monitoring Fe levels in women, particularly during menopause, which may reduce the risk of AD and related cognitive impairment.

A previous study showed low levels of erythrocyte Mn in women AD patients [65]. This suggests that Mn homeostasis might interact with sex-specific biological factors, such as the role of estrogens in metal transport and antioxidant defenses.

### 4.3. Women’s Vulnerability in Other Neurodegenerative Diseases

Various studies suggested a link between specific metal exposures and neurodegenerative diseases. However, there is only a little evidence on the effect of sex on the risk of metal-induced neurodegenerative diseases as reported in the current literature. A recent study conducted a meta-analysis and systematic review to analyze epidemiological evidence of associations between metal exposure and Parkinson’s disease [105]. Similarly, associations were observed between Mn accumulation and Mg, AD, Parkinson’s disease, Huntington’s disease, and amyotrophic lateral sclerosis (ALS) [106]. In the case of ALS, metal exposure was highlighted in several studies as a potential risk factor. For instance, in an ecological study of 62 ALS patients, copper (Cu) was found to be directly correlated with ALS in both sexes [107]. An interesting observation of the study was a higher correlation in women compared to men (0.78 vs. 0.60). In another case–control study conducted in New England (N = 109) from 1993 to 1996, both blood and bone lead were linked to ALS, although this study did not show any effects of sex [108]. In a multicenter prospective cohort Multi-Ethnic Study of Atherosclerosis (MESA), As, Cd, Co, Cu, W, U, and Zn were individually associated with dementia, although no significant differences between men and women were reported [109]. Even though very little evidence of metal-induced neurodegenerative diseases were found in the literature, scientists indicated specific mechanisms that could put women at higher risk when exposed to metals. One possible mechanism is the stronger immune system of women than men, which may result in higher accumulation of amyloid plaques, one potential risk factor of AD/ADRD [110]. Other possible mechanisms of the neurotoxicity of metals, such as oxidative stress, mitochondrial dysfunction, activation of microglial cells and inflammation, or promotion of α-synuclein aggregation and fibril formation, are often higher in older women, putting them into higher risk of neurodegenerative diseases [105,111,112].

### 4.4. Possible Mechanisms Linking Metal Exposure, Sex Differences, and Cognitive Impairment

Emerging evidence suggests that heavy metals such as Pb, Cd, As, Cu, and Fe induce cognitive decline and neurodegenerative diseases like AD/ADRD. While many studies cited in this review showed women’s vulnerability to metal exposure-induced ADRD, some studies could not ascertain that. Nonetheless, generally, women are much more prone to ADRD than their men counterparts as evident in the majority of the studies. The observed sex-specific vulnerability, particularly among women, may be influenced by differences in hormonal regulation, antioxidant capacity, and metal metabolism.

#### 4.4.1. Neurotoxicity and Oxidative Stress

Chronic exposure to Pb, Cd, and As generates reactive oxygen species (ROS), leading to neurotoxicity, which is a primary contributor to cognitive impairment in older adults [113]. Additionally, an imbalanced Cu/Zn molar ratio has been associated with oxidative stress, contributing to AD. Several studies presented in this review provide evidence to support that relatively poor antioxidant defense may be a possible mechanism that puts older women at a higher risk of getting AD/ADRD. Furthermore, estrogen has a neuroprotective role that declines with age, making post-menopausal women more susceptible to oxidative damage [114].

#### 4.4.2. Hormonal Influence on Metal Metabolism

**Figure 2:**
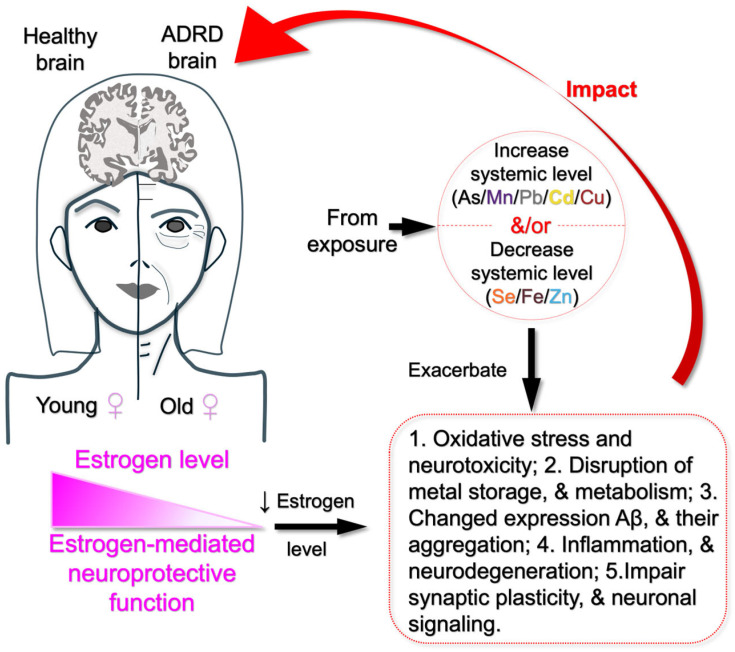
Pathway linking metal exposure to estrogen decline and ADRD risk in older women. Summarized possible mechanisms underlying the findings from the cited literature, suggesting that estrogen decline in older women increases their vulnerability to oxidative stress, neuroinflammation, disruption of metal storage and metabolism, neurodegeneration, and impaired expression and aggregation of amyloid-beta. These effects are further exacerbated by elevated levels of toxic metals (As, Mn, Cu, Cd, and Pb) and decreased levels of essential metals (Se, Zn, and Fe) due to environmental exposure, ultimately contributing to ADRD in older women.

Sex hormones such as estrogen impact the transport and storage of essential metals like Fe, Cu, Zn, and Se [115,116,117]. However, declining levels of estrogen with increasing age may have a negative effect on body’s ability to regulate these metals, which may lead to metal accumulation or deficiency. Cd disrupts endocrine function, and by mimicking estrogenic activity, it may contribute to greater Cd retention in women, worsening neurotoxic effects [118]. Similarly, since estrogen influences Mn and Se levels [117,119], lower levels of estrogen with aging may affect the essential metals in the body. As a result, deficiencies in these metals disproportionately affect cognitive function, more likely in females (Figure 2).

#### 4.4.3. Inflammatory Pathways and Neurodegeneration

Pb-, Cd-, and As-induced toxicity, among others, triggers inflammatory cascade, contributing to neuronal death, which is a hallmark feature of AD pathology [10]. Due to aging and lower levels of estrogen, elderly women may have a higher baseline level of inflammation that could explain their increased susceptibility to metal-induced cognitive decline.

#### 4.4.4. Metal-Induced Protein Aggregation

Abnormal protein aggregation, particularly of β-amyloid (Aβ) and tau, has been implicated in AD pathology. Metal ions of Cu, Fe, and Zn can interact with Aβ peptides, promoting aggregation and plaque formation [120]. Some of the studies, presented in this review showed a positive correlation between an increased Cu/Zn molar ratio and AD in elderly women, which may be due to the disrupted metal homeostasis and its putative contribution to the Aβ peptides aggregation and plaque formation. Moreover, women are more susceptible to AD if they have elevated plasma levels of Fe due to hormonal influences on iron metabolism.

#### 4.4.5. Potential Protective Roles of Zn and Se

While heavy metal accumulation may result in neurotoxicity, deficiencies in essential trace elements like Zn and Se have also been implicated in cognitive decline. Zn plays a crucial role in synaptic plasticity neuronal signaling, and neuroprotection and its deficiency leads to the development of neurodegenerative disease, including AD [121] On the other hand, Se is involved in antioxidant defense and brain function, and a lower level of Se has been strongly linked to AD [53,122]. Aging women are prone to these trace metal deficiencies due to the combination of hormonal changes, lower absorption rates, higher oxidative stress, dietary differences, chronic inflammation, and gut health issues [123]. These factors, therefore, may contribute to women’s vulnerability to poor cognitive function, potentially leading to the development of AD.

### 4.5. Limitations

The current review encountered several methodological limitations, primarily due to weaknesses in the epidemiological design or sample size. Our inclusion criteria limited our selection to articles that were indexed, available online, and written in English. The research team was not equipped to review articles that are not in English. Furthermore, the majority of the AD/ADRD papers that can reach the largest possible audience across the globe are written in English. As a result, studies published in some other languages, such as Chinese, Spanish, or Portuguese, were not considered, which may have excluded important articles and narrowed the scope of our review.

Most of the studies included in our review were cross-sectional, with only a small number of cohort and case–control studies that met our selection criteria. Cross-sectional studies do not provide information about temporality; however, it is very unlikely that metal-induced health outcomes occurred before exposure to metals during adulthood. Another limitation was the small sample size observed in multiple studies, producing low statistical power for detecting the effect of high versus low exposure impact or for identifying differences between men and women regarding AD/ADRD outcomes. Additionally, our review did not focus on genetic factors that may put some women at higher risk of AD/ADRD. This was beyond the scope of the review.

Therefore, future investigations should consider prospective epidemiological designs with larger sample sizes to establish causality between metal exposure and AD/ADRD outcomes, particularly in women. Finally, the great diversity of biomarkers used to assess metal exposure (serum, plasma, whole blood, hair, urine, etc.) in various studies made it difficult to compare the results, making it challenging to draw inferences.

## 5. Conclusions and Future Directions

Despite some methodological challenges, our review identified Zn and Fe as the potential neuroprotective agents for AD/ADRD-related outcomes. In contrast, Cd, Pb, and As primarily exhibited neurodegenerative effects in AD/ADRD patients. Se displayed both positive and negative effects. However, several studies presented contradictory information indicating that heavy metals could have protective, adverse, and/or no effects on neurocognition in older adults, particularly in older women. Moreover, only a few studies examined and published results based on sex (male vs. female), which posed a major challenge in our review. The interplay between these metals and their cumulative neurotoxic effects highlights the need for sex-specific research frameworks to address women’s unique susceptibilities. Understanding these dynamics is essential for developing tailored interventions and prevention strategies targeting metal dysregulation in AD/ADRD. Therefore, we recommend large randomized controlled trials (RCTs) or prospective cohort studies emphasizing sex differences to further our understanding of the role of metals in the development of AD/ADRD. The findings from larger epidemiological studies may help our policymakers and interventionists develop therapeutic or behavioral interventions for preventing the worsening of AD/ADRD symptoms in older women around the world.

## Figures and Tables

**Table 2:** Epidemiological studies describing adverse effects and no effects of metals with AD/ADRD outcomes.

Reference	Metal(s) and Common Source	Diagnosis	Major Findings Related to Sex Differences
Ashraf et al. (2019) [46]	Plasma Zn; Food/ processed food	MMSE, CDR, and APOEε4-positive (n, %)	Zn was higher in AD. Sex was not statistically significant.
Duan et al. (2024) [64]	Ca, Zn, Fe, and Pb; Not mentioned	Nine standardized cognitive tests	No significant difference in sex. Higher levels of Ca (*p* = 0.025) were associated with lower cognitive function. Zn, Fe, and Pb were not associated.
Koseoglu et al. (2021) [52]	Nail and hair As and Se; Ground water	MMSE	As and Se levels were not associated with sex. Positive correlation between As and Se levels within the AD group (*p* < 0.01).
Fathabadi et al. (2018) [38]	Blood Pb; Industrial pollutants	MMSE	BLL was significantly higher in the patients group. Sex was not statistically significant. BLL was lower in women (*p* = 0.01).
Giacconi et al. (2019) [47]	Plasma Cu; Not mentioned	MMSE	No significant differences were found for sex. Significantly higher Cu levels among AD participants.
Gu et al. (2021) [54]	Blood As and Se; Not mentioned	MMSE	As and Se were associated with the increased risk of CI (*p* < 0.05). Sex was not statistically significant.
Huang et al. (2022) [55]	Blood Cd; Food	AFT, CERAD, and DSST	High-level Cd significantly was associated with low cognitive performance. Sex was not correlated.
Yu et al. (2023) [49]	Whole-blood Zn; Drinking water	ADL and MMSE	Higher Zn increased the risk of MCI, especially among women (*p* < 0.05).
Jouini N et al. (2021) [56]	CSF Fe; Not mentioned	Brain imaging and MMSE	Fe significantly higher in the AD group (*p* < 0.001). No difference was observed in women.
Zhang et al. (2022) [18]	Plasma Cu and Cd; Food, water, and industrial pollutants	MMSE	Significant positive correlation between the risk of CI and Cu and Cd (*p* < 0.05). Sex was also statistically significant, and most CI participants were female.
Koc et al. (2015) [57]	Hair Cu, Mn, and Mg; Not mentioned	MMSE	No significant variations based on sex. Significantly higher hair Cu and Mn levels found in AD patients (*p* < 0.05). Mg showed no effects on AD.
Lui H et al. (2021) [15]	Blood Cd; Air, water, soil, and food	Nine standardized cognitive tests	Higher Cd levels were significantly associated with greater cognitive decline. Sex wasstatistically significant (*p*< 0.05).
Li H et al. (2018) [58]	Blood Cd; Not mentioned	CERAD, Word List Learning Test, the CERAD Word List Recall Test, AFT, and DSST	Higher level of blood Cd was associated with worse cognitive function. Sex was significant (*p*< 0.05).
Liu Q et al. (2024) [44]	Blood Pb; Not mentioned	MMSE	Significant association with Pb and CI (*p* < 0.05). Sex was significant (*p* < 0.001), and most CI individuals tended to be women.
Power et al. (2014) [17]	Bone Pb; Not mentioned	Telephone-based cognitive assessment	Long-term cumulative Pb exposure weakly associated with faster cognitive decline among women.
Min J et al. (2016) [50]	Blood Cd; Air and industrial pollutants	NHANES (1999–2004)	Female participants (0.50 μg/L) had higher blood Cd levels. Significant association between high Cd levels and risk of AD mortality (*p* = 0.0684).
Wang et al. (2024) [66]	Blood Cd; Air	CERAD, Word Learning Test, AFT, and DSST	Higher blood Cd was associated with lower cognitive scores. Women group did not show any association.
Park et al. (2019) [59]	Brain Fe; Not mentioned	CDR, GDS, MMSE, and brain MRI	In the motor cortex, there was higher Fe among AD patients. No difference was observed in women.
Peng Q et al. (2017) [51]	Blood Cd; Not mentioned	NHANES 1999–2006	Women (0.52 ng/mL) had significantly higher blood Cd level. Increased blood Cd was associated with AD mortality (*p* = 0.04).
Rozzini et al. (2018) [60]	Serum Cu; Not mentioned	MMSE	Higher Cu in MCI and dementia was due to AD (*p* < 0.0001). Women showed higher total Cu levels in both groups.
Socha et al. (2021) [36]	Serum Cu and Zn; Food	MMSE	The Cu/Zn molar ratio was significantly higher among women with AD.
Sternberg et al. (2017) [61]	Serum Fe; Not mentioned	MMSE, CDR, CDR-SOB, neuroimaging, and clinical data	Fe among AD patients was significantly higher (50%) compared to controls (*p* = 0.004). No difference was observed in women.
Wang et al. (2021) [19]	Hair As; Environment	MMSE	Women had a significantly higher prevalence of CI. Positive correlation between As and CI (OR= 1.84, *p* < 0.05).
Wang et al. (2024) [67]	Plasma Ca; Environment	MMSE	Plasma Ca was positively correlated with CI risk. Women group did not show any association.
Qing et al. (2024) [16]	Blood Bb and urinary Cd and Pb; Food	MoCA	Pb and Cd levels were significantly correlated with MoCA score. Sex was significant (*p* < 0.05), but women group did not show any association.
Yang et al. (2018) [33]	Whole-blood Se and urine As; Drinking water	CDR and MMSE	Women were significantly more likely to have AD. Significant association with both Se and As with AD risk (OR = 1.9, *p* < 0.05).
Fu et al. (2024) [45]	Blood Pb, Cd, and Cu; Air, water, and food	IRT, DRT, AFT, and DSST	High levels of Pb, Cd, and Cu were correlated with cognitive function. Female group had higher blood Pb level (2.09 µg/dL).

**Table 3:** Epidemiological studies describing the beneficial effects of metals with AD/ADRD outcomes.

Reference	Metal(s) and Common Source	Diagnosis	Major Findings Related to Sex Differences
Ashraf et al. (2019) [46]	Plasma Fe; Food/processed food	MMSE, CDR, and APOEε4-positive (n, %)	Fe was lower in AD. Sex was not statistically significant.
Cardoso et al. (2017) [53]	Erythrocytes, serum, and CSF Se; Not mentioned	GDS and MMSE	Erythrocyte Se levels were significantly lower in AD (*p* < 0.05). No significant differences were found for sex.
Cheng et al. (2022) [63]	Urine Se; Food	MMSE	Se was positively associated with MMSE scores and cognitive function. Lower MMSE scores were found in women (*p* < 0.01).
Duan et al. (2024) [64]	Se, Cu; Not mentioned	Nine standardized cognitive tests	No significant difference in sex. Higher levels of Se (*p* = 0.007) and Cu (*p* = 0.048) were associated with better cognitive function in the elderly.
Giacconi et al. (2019) [47]	Plasma Zn; Not mentioned	MMSE	No significant differences were found for sex. Significantly lower Zn among AD patients.
Gong et al. (2021) [34]	Serum Fe; Not mentioned	DSST, AF, CERAD-DR, and CERAD-WL	Sex was not significant. Inverse dose–response relationship between serum Fe and CI.
Hare et al. (2016) [65]	Serum and erythrocyte Mn; Not mentioned	AIBL	Lower erythrocyte Mn (*p* = 0.053) in women. Serum Mn was decreased in AD compared to HC (*p* < 0.001).
Yu et al. (2023) [49]	Whole-blood Se; Drinking water	ADL and MMSE	Lower Se in MCI, especially among women (*p* < 0.05).
Jouini N et al. (2021) [56]	CSF Ca; Not mentioned	Brain imaging and MMSE	Ca was significantly lower in the AD group (*p* < 0.001). No difference was observed in women.
Koc et al. (2015) [57]	Hair Se and Zn; Not mentioned	MMSE	No significant variations based on sex. Significantly lower hair Se and Zn levels were found in AD patients (*p* < 0.05).
Nascimento et al. (2021) [35]	Plasma and erythrocyte Se; Food	NINCDS criteria by geriatrician and MMSE	Elderly with AD presented lower Se concentrations (*p* = 0.028). Odds ratio between men and women was 0.51.
Wang et al. (2024) [66]	Blood Se; Air	CERAD, Word Learning test, AFT, and DSST	Se has protective effect on cognitive function. Women group did not show any association.
Rembach et al. (2014) [48]	Serum Zn; Not mentioned	MMSE	Non-significant lower Zn levels in AD women. Serum Zn levels were significantly lower in AD patients compared to healthy controls (*p* = 0.001).
Socha et al. (2021) [36]	Serum Se and Zn; Food	MMSE	Significantly lower serum Se and Zn in AD patients, with women showing even lower (*p* < 0.05).
Xu et al. (2018) [62]	Serum Mg, Ca, Fe, Cu, Zn, and Se; Not mentioned	MMSE	Among female patients, none of the measured serum elements of serum Mg, Ca, Fe, Cu, Zn, Se differed in their plasma concentrations between the AD and control groups. Low levels of Zn and Fe were found in women participants.
Wang et al. (2024) [67]	Plasma Mn, Fe, and Zn; Environment	MMSE	Plasma Mn, Fe, and Zn levels were negatively correlated with CI risk. Women’s group did not show any association.

## Data Availability

Data are contained within the article.

## Abbreviations

The following abbreviations are used in this manuscript:

AD	Alzheimer’s disease
ADL	Activities of daily living scales
AFT	Animal Fluency Test
AIBL	Australian Imaging Biomarkers and Lifestyle Flagship Study of Ageing
AD/ADRD	Alzheimer’s disease and related dementias
APOE4	Apolipoprotein E4
BLL	Blood lead level
CI	Cognitive impaired
CN	Cognitive normal
CDR	Clinical Dementia Rating
CDR-SOB	Clinical Dementia Rating—Sum of Boxes
CERAD	Consortium to Establish and Registry for AD Test
CERAD-DR	Consortium to Establish and Registry for AD Test—Delayed Recall
CERAD-WL	Consortium to Establish and Registry for AD Test—Word Learning
CSF	Cerebrospinal fluid
DSST	Digit Symbol Substitution Test
DRT	Delayed Recall Test
GDS	Geriatric Depression Scale
HC	Healthy control
HE	Healthy elderly
HICs	High-income countries
IRT	Item response theory
LCP	Low cognitive performance
LMICs	Low- and middle-income countries
MCI	Mild cognitive impairment
MMSE	The Mini-Mental State Examination
MRI	Magnetic resonance imaging of the brain
MoCA	Montreal Cognitive Assessment
NHANES	National Health and Nutrition Examination Survey
NINCDS	National Institute of Neurological and Communicative Disorders and Stroke
RCTs	Randomized controlled trials
ROS	Reactive oxygen species
